# Genome-wide analysis of chemically induced mutations in mouse in phenotype-driven screens

**DOI:** 10.1186/s12864-015-2073-4

**Published:** 2015-10-26

**Authors:** Denis C. Bauer, Brendan J. McMorran, Simon J. Foote, Gaetan Burgio

**Affiliations:** Digital Productivity, CSIRO, 11 Julius Av, Sydney, 2113 Australia; Australian School of Advanced Medicine, Macquarie University, 2 technology place, Sydney, 2109 Australia; John Curtin School of Medical Research, The Australian National University, GPO Box 334, Canberra, 2600 Australia

**Keywords:** ENU, NGS, Variants, Forward genetics

## Abstract

**Background:**

N-ethyl-N-nitrosourea (ENU) mutagen has become the method of choice for inducing random mutations for forward genetics applications. However, distinguishing induced mutations from sequencing errors or sporadic mutations is difficult, which has hampered surveys of potential biases in the methodology in the past. Addressing this issue, we created a large cohort of mice with biological replicates enabling the confident calling of induced mutations, which in turn allowed us to conduct a comprehensive analysis of potential biases in mutation properties and genomic location.

**Results:**

In the exome sequencing data we observe the known preference of ENU to cause $A:T\Rightarrow G:C$ transitions in longer genes. Mutations were frequently clustered and inherited in blocks hampering attempts to pinpoint individual causative mutations by genome analysis only. Furthermore, ENU mutations were biased towards areas in the genome that are accessible in testis, potentially limiting the scope of forward genetic approaches to only 1–10 % of the genome.

**Conclusion:**

ENU provides a powerful tool for exploring the genome-phenome relationship, however forward genetic applications that require the mutation to be passed on through the germ line may be limited to explore only genes that are accessible in testis.

**Electronic supplementary material:**

The online version of this article (doi:10.1186/s12864-015-2073-4) contains supplementary material, which is available to authorized users.

## Background

Chemically-induced mutations in the model organisms and screening the resulting phenotype has proven to be a powerful tool for annotating gene function and to uncover the biological mechanisms of diseases [1, 2].

Malaria remains the third lethal disease and affect mostly children and non-immune adults [3]. The rapid spread of drug-resistant malaria parasites remains a deep concern [4]. A novel strategy is urgently needed to combat the rapid development of drug-resistant parasites. Genetic diseases affecting the red blood cell are common in populations resident in malarial endemic regions [5]. Genetic mutations in HbS, HbC, HbE and G6PD or both and *β*-thalassaemia are protectives as heterozygotes against malaria [6]. These genetic mutations that are protective against malaria could be described as endogenous antimalarial therapies and the parasite has difficulty in developing resistance against the host [7]. The aim of this project was to uncover novel host-encoded targets by producing lines of mice that survive an otherwise lethal *P. chabaudi* infection, but also to screen for abnormal red blood cells count as most human mutations giving rise to resistance have an abnormal red cell phenotype in either the homozygous and/or heterozygous state [6]. N-ethyl-N-nitrosourea (ENU) is the mutagen of choice for inducing random mutations into the mouse or zebrafish genomes (see [8, 9]).

ENU causes alkylation of nucleotides with subsequent mispairing and ultimately base-changes after replication. Justice et al. [10] reported that the most common mutations induced by ENU in the mouse genome are A:T to T:A transversions at the DNA level, and at the protein level are inducing mainly missense mutations, but also non-sense mutations, as well as alternative spicing mutations, gain-of-stop mutations, intronic mutations or noncoding mutations causes amino-acid changes, folding or expression of the protein [2].

ENU was used to induce new mutations in a strain of mouse that is normally susceptible to the rodent malaria parasites. The mice were challenged with *P. chabaudi* or *P.berghei* parasites and selected, as animals of interest, those that survived to the infection or displayed an abnormal red blood cell count. As is the case with the natural mutants found in endemic malaria infection areas, these induced mutations resulted in mice surviving a malarial infection and marked possible novel host targets. This property can be used to randomly introduce single base-pair mutations, thereby surveilling the genome’s function and potential in an unbiased fashion.

However, a recent study sequencing *Toxoplasma gondii* genomes after ENU mutageneis revealed that the introduced changes were not randomly distributed in the genome [11]. While they confirmed Justice et al. [10] observation of ENU’s proclivity for inducing mutations at A/T base pairs (78.6 %), they also observed a higher transition (ti) compared to transversion (tv) rate (ti/tv ratio 1.20) [11]. Similar biases may be present in ENU-based phenotype screens in mice, as indicated by Barbaric et al. [8], who did a meta-analysis of all reported ENU mutations from targeted gene studies. They observed that ENU targeted genes had higher coding sequence length, higher exon number and had a higher GC content than the average for the mouse genome. Furthermore, ENU mutations were often directly flanked by G or C nucleotides.

Here we investigate the observations made by Barbaric et al. on a sequencing cohort of ENU treated mice. Specifically, we discuss how to distinguish ENU-induced variants from lab-strain specific, germline single nucleotide polymorphisms (SNPs) and sporadic somatic variants. Furthermore, we investigate biases in the ENU variant distribution due to DNA sequence motifs or open chromatin structure in the targeted tissue.

## Results and discussion

### Coverage and ENU statistics

We received on average 74 million reads (ste = 2531660) from exome sequencing of which on average 94.8 % (ste = 0.2) mapped to the genome with 90.4 % (ste = 0.3) being paired using BWA and 97.9 % (ste = 0.2) mapping with 93.2 % (ste = 0.2) being paired using BOWTIE2. The mean coverage over the captured exons was 53.3 % (ste = 20.5) and 56.2 % (ste = 21.6), respectively, with on average 75.0 % (ste = 11) and 77.4 % (ste = 10.5) of bases being covered with 25 × (see Additional file 1: Table S1).

Table 1 shows that on average 13,775,275 variants are called by the various variant calling methods for all mice. We removed the likely false positives by filtering out discordant variants (not present in all offspring from a founder) and non exclusive variants (variants also present in other founders) to only on average between 39 and 66 variants per method (P1-P4) and for each mouse. The rational for this is that the ENU variants are by probability unlikely to be identical in other independent founders line.

**Table 1:** The table shows the variant numbers identified by the different methods through the different filtering steps. Note, mean raw calls per mouse is not available for P4 as GATK calls jointly over all mice. Pass QC involves filtering for olfactory genes, known Sanger variants and PASS flag from GATK

	P1	P2	P3	P4
Mean raw calls	13,775,275			
Mean raw calls	66,493	71,705	74,093	NA
per mouse				
Total raw calls	145,484	131,926	124,788	54,698,903
(for 53 founders)				
Mean filtered	39	44	41	66
SNVs per mouse				
Mean combined	111			
filtered SNVs				
Mean agreeing	54			
SNVs				
Mean pass QC	21			
Total ENU mutation	1281			
(for 53 founders)				

For the next filtering step, we took the union over the four methods resulting in an average of 111 variants per mouse. We aimed to reduce the false positive rate further by requiring two or more methods to support the variant call, which reduces the set to 79 variants per mouse. We further reduced the set by removing variants that are not consistent with the genetics of the phenotype segregation resulting in an average of 56 variants per mouse. The ENU candidate variants were further refined by filtering out variants that did not pass the GATK quality control, which assesses alignment statistics (e.g. read depth), resulting in 21 variants on average per mouse and 1281 variants in total over all 53 founders. This very stringent filtering regime was designed to reduce the false positive rate with already 39 identified variants confirmed as ENU mutations (data not shown).

**Fig. 1:**
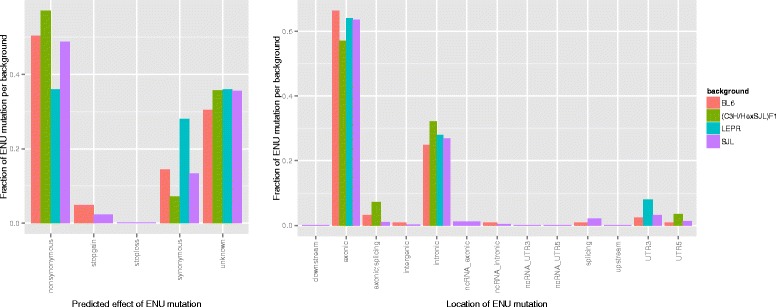
Functional annotation of ENU mutation per background

Mice with a SJL background had 60 % more predicted ENU mutations than C57BL/6 (*t*-test *p*-value = 0.01575), which makes the background the determining factor for ENU mutation number over sequencing depth, sequencing provider or capture technology (Additional file 1: Figures S1–S4) potentially resulting from a elevated dose of ENU injection for SJL mice. Locations with ENU mutation had lower coverage than the average in the genome (52 vs 57). This was statistically significant (log-transformed *t*-test *p*-value =2.2·10^−16^) reduction but can be explained with them predominantly being located in exonic regions (see Fig. 1), which tend to have less sequence duplications or repetitive regions and as a result have consistent coverage. The correlation coefficient between overall coverage and ENU mutation coverage was 0.39 and *p*-value =2.2·10^16^ (Additional file 1: Figure S5). 40 % to 60 % of the putative ENU variants were nonsynonymous and on average less than 20 % were synonymous with the rest were unknown as they were located intronic or in the 3’UTR (see Fig. 1).

### ENU induced variants have different profiles than germline and somatic variants

#### ENU mutations are enriched in longer genes

As reported by Barbaric et al. [8], genes targeted by ENU tend to have a higher coding sequence length than the average mouse gene. We also observe this reported bias in our dataset with genes containing one or more ENU mutations having an average of 88,499 bp compared to the average gene length of 26,550 bp in the mouse genome (log-transformed *t*-test *p*-value =2.2·10^−16^). This can be explained by the simple fact that the longer a gene the higher its probability of incurring a mutation. However contrary to the results by Barbaric et al., the GC content in our dataset is significantly lower than the mouse genome average (45.6 % vs 46.1 % *p*-value = 0.006). To avoid selection bias, we investigated the G1 generation only as no phenotype selection was performed at this stage of breeding (653 variants in 21 founders). However, the results for G1 mirrored the full dataset (see Table 2). We also compared two publicly available datasets of reported ENU mutations. Here we also found a significantly increased gene length for ENU mutations in Mutagenetix (*p*-value =2.2·10^−16^) and Phenomics (*p*-value =2.2·10^−16^), respectively, while the GC content was found not to be significantly different from the mouse average (see Table 2).

**Table 2:** Mean of GC and gene length Note, not all identified variants are located in annotated genes

	Number of genes	CG	Gene length
Mouse genome	43629	46.1602	26550.38
ENU all	1186	45.6114	88498.69
ENU G1	613	45.1885	94356.03
Mutagenetix	19491	46.1220	49908.29
Phenomics	20496	46.1544	48533.53

#### ENU mutations are dominated by $A:T\Rightarrow G:C$ transitions

**Fig. 2:**
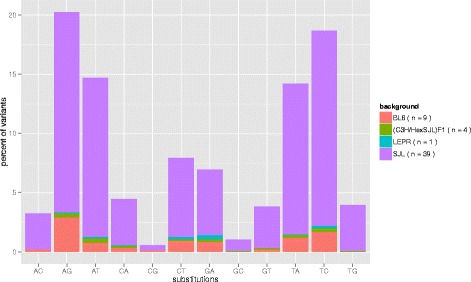
Substitution frequency

Next we investigated the base pair substitution rate in our data set. As shown in Fig. 2, 20 % of the mutations were $A\Rightarrow G$ transitions closely followed by $T\Rightarrow C$ transitions and 15 % are $A\Rightarrow T$ and $T\Rightarrow A$ transversions. We observed a similar distribution (correlation coefficient cc = 0.986, *p*-value =6.1·10^−10^) for the G1 dataset (see Additional file 1: Figures S6 and S7) as well as the Mutagenetix (cc = 0.993, *p*-value =1.3·10^−10^) and Phenomics (cc = 0.992, *p*-value =2.8·10^−10^) datasets.

#### ENU mutations have no flanking motifs

Barbaric et al. [8] reported a significant overrepresentation of G or C at either side of the mutation. To investigate this, we extracted the flanking 10 bp sequences from the reference genome and visualised this potential ENU motif using WEBLOGO [12]. As expected we found an overrepresentation of A and T bases, as they make up around 70 % of the targeted bases (Fig. 2), resulting in an overall information content of less than 0.5 at the central position. We did not observe any other overrepresented base in the 10 bp flanking the ENU mutation. We then stratified by targeted base to investigate if there was a targeted base specific motif. Due to the lower number of involved sequences we saw certain overrepresented bases however, they are also present in randomly selected regions (Additional file 1: Figure S8). Again, removing bias from phenotype selection we focussed on the G1 mice only but did not observe a motif here either (Additional file 1: Figure S9). Finally, to remove residual bias from the mouse strain, we focused on mice with SJL (39 founders) and (C3H/HexSJL)F1 (four founders) background as we had a strain specific reference genomes for these stains before ENU mutations were introduced. The logos generated from the 1116 mutations in SJL mice represent the logo obtained over all and investigating the 27 mutations for (C3H/HexSJL)F1 did not produce a reliable motif due to the small numbers investigated. Overall, we did not observe a flanking motif for ENU mutations in our dataset.

### ENU mutation distribution

**Fig. 3:**
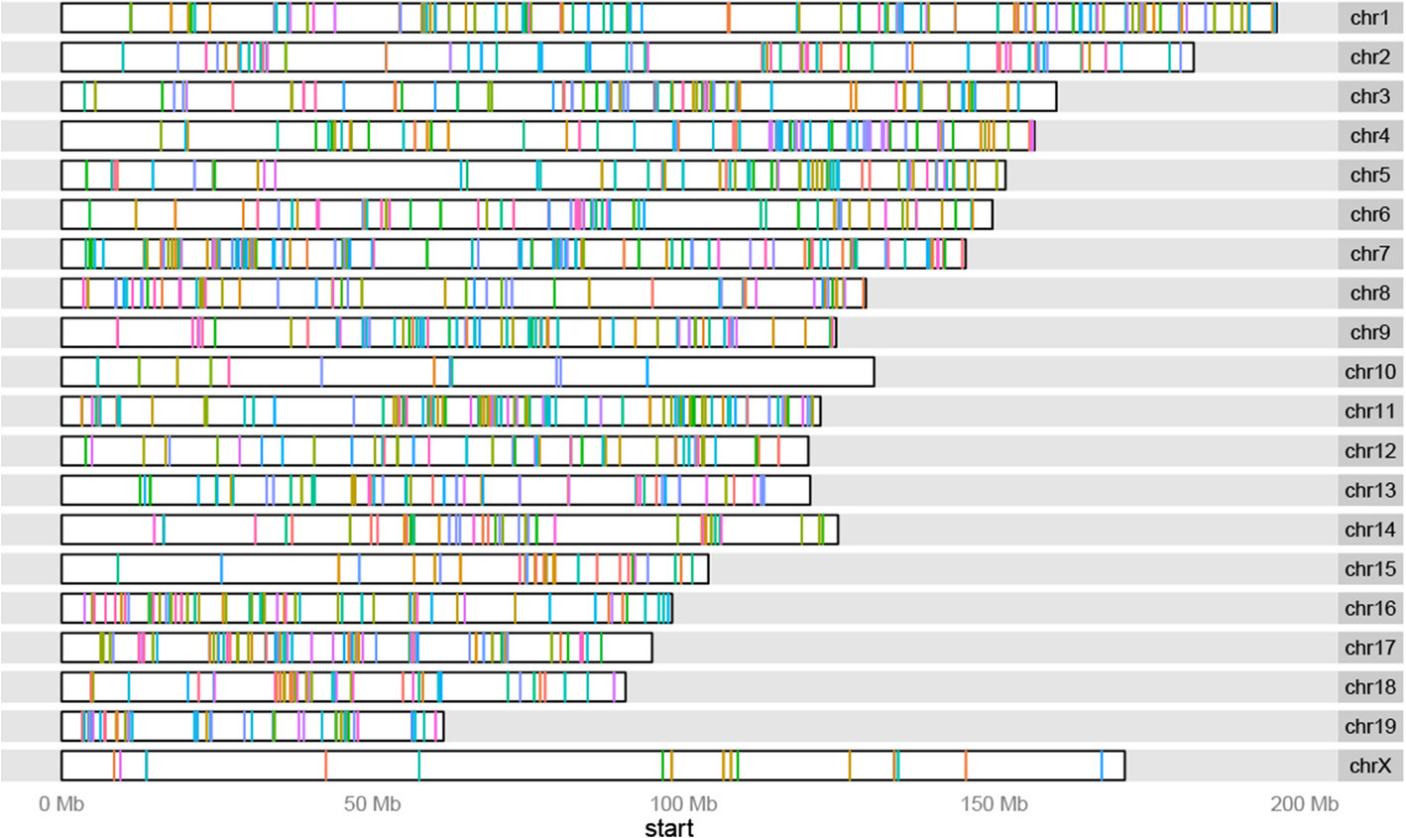
ENU variants cluster within each mouse. Variants are coloured by founder to display clear blocks of inheritance within mice

We plotted the ENU mutation locations along the genome to determine whether they are uniformly distributed or if there are factors other than gene length biasing the distribution. Figure 3 visualises all ENU mutations colour coded by the founder they occur in. Overall ENU mutations were not uniformly distributed. This becomes even more apparent when plotting the mutations reported in the larger Mutagenetix and Phenomics datasets (Additional file 1: Figures S13 and S14), where there are areas of depletion.

One explanation for this could be that due to the subsequent phenotype screening it is likely that ENU mutations are located in protein coding genes, which in turn are non-uniformly distributed in the genome. To test this, we count how many genes have at least one ENU mutation when randomly shuffling ENU locations (1000 times). We observe on average 24.71 genes to be targeted by the real ENU mutations per mouse compared to 10.95 when randomly selecting locations, under the Poisson distribution seeing this many genes given the lambda of 11.13 is highly unlikely (0.0002), which is not surprising give the sequencing assay. However, when restricting the shuffling to gene locations only we observe on average 25.45 genes, though not statistically significant (Poisson 0.44), ENU mutations seem to be more clustered thereby targeting less genes. A density plot (Additional file 1: Figure S16) of targeted genes for ENU mutations *versus* random locations in the exome shows that the underlying distributions could be different specifically in the tail. We therefore hypothesise that ENU mutations are not independent and there are additional limiting factors other than gene location.

### ENU mutation are inherited in blocks

Putative ENU mutations appeared to be clustered by founder (see Additional file 1: Figure S15 for an image per founder). The average distance between ENU mutations in a founder is 44 million bp (std = 38 million bp). To determine the significance of these observations in our dataset, we calculated an empirical *p*-value by shuffling the founder label for every ENU mutation 10,000 times and recalculated the distance thereby testing *p*-values down to 0.0001. The resulting *p*-value was 0.0003 with average distance 48 million bp (std = 37 million bp). This suggests that ENU mutations are inherited as blocks.

### ENU mutation distributions biased towards open chromatin areas

ENU mutations occur systemically, however, only the mutations introduced in the germline are passed on to the next generation. A hypothesis is hence that ENU mutations are biased towards the parts of the genome that are accessible in the testis, which would limit the functional assessments to testis-specific genes.

**Fig. 4:**
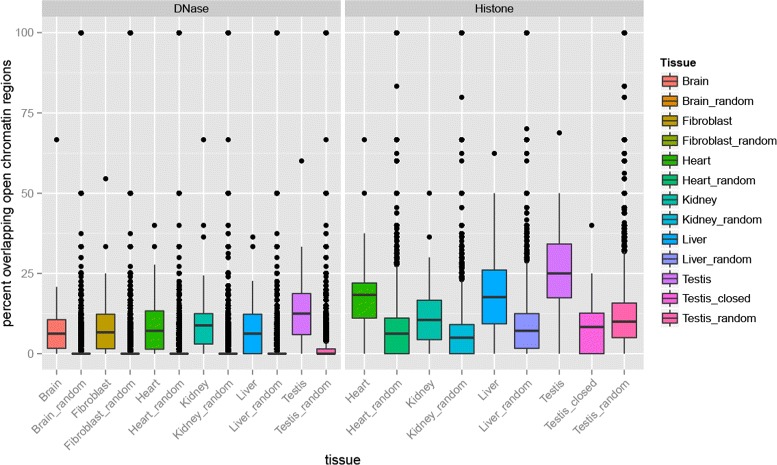
Variants are enriched in open chromatin areas of testis. *left*: Percentage of ENU variants overlapping DNase hypersensitivity areas in testis and other tissues as well as random genomic regions. *right*: Percentage overlap with open chromatin area as flagged by Histone modification marks from ChipSeq experiments

To test this hypothesis we investigated whether ENU mutations were enriched in open chromatin areas of testis as determined by DNase hypersensitivity sequencing data (DNase-Seq) and compared this against the DNase-Seq regions of other tissues as well as randomly sampled regions of the same size. As shown in Fig. 4, we observed a significant enrichment of ENU mutations in testis DNase-Seq regions compared to brain (two-sided Wilcox rank sum test with fdr correction *p*-value = 0.0052), fibroblast (0.0224), heart (0.0487), kidney (0.0358), liver (0.0056). As there are more base pairs accessible in testis (38,505,954 bp in over 81,467 regions) compared to other tissue (average 29,442,913 bp and 68,266 regions), ENU variants have a higher probability to fall into these regions by chance. To investigate this, we randomly sampled 1000 times regions of the same size and chromosome distribution as the testis dataset and compared these values to the observed overlap in testis. As shown in Fig. 4, the overlap with the open chromatin areas in testis were significantly different to that of the randomly generated dataset (< 2·10^−16^) substantiating the bias of ENU mutation towards accessible areas. To demonstrate that this was not due to properties of the testis open chromatin region we also tested the observed overlap to the randomly sampled genomic regions for all other tissues and observed consistently a significantly lower overlap in the randomly sampled regions datasets (< 2·10^−16^).

As an orthogonal test, we used histone marks to determine the open chromatin areas. As reported by Shlyueva et al. [13], histone marks H3K4me1 and H3K27ac encode active promoters or enhancers, while H3K27me3 flags inactive areas. From this information we determined the open chromatin areas (see “Methods”), and again compared testis against other tissues as well as randomly sampled testis-like regions. As shown in Fig. 4, we again found a significant enrichment of ENU mutations in testis DNase-Seq regions compared to heart (0.00279), kidney $\left (4.1\cdot 10^{-6}\right)$, liver (0.01799) and random sampled regions $\left (3.4\cdot 10^{-12}\right)$. Finally, we saw a significant depletion of ENU mutations in areas marked by H3K27me3 to be inactive in testis tissues compared to open chromatin testis $\left (3.1\cdot 10^{-8}\right)$ or random sampling of testis regions (0.03476).

## Conclusions

ENU treatment is most effective in the accessible areas of the genome. Phenotype screens, which require the ENU mutation to be passed on through the germ line are hence limited to areas accessible in testis. This limits the explorable area to only 1–10 % of the genome depending on which proxy for openness is used (3.3·10^8^ bp Histone marks and 3.9·10^7^ bp DNase of the 2.8·10^9^ bp). We provided a list of genes potentially not targeted by ENU mutation (Additional file 2). Indeed when comparing to the Mutagenetix and Phenomics dataset only between 5–6 % of all mouse genes are repeatedly (twice) targeted by ENU mutations and the overlap with closed chromatin areas in testis is very low (jaccard statistic 8.6·10^−5^ and 0.0001, respectively).

## Methods

### ENU treatment

A dominant large scale ENU mutagenesis screen was performed. SJL/J and C57BL/6 and BKS.Cg-Dock7^m^ +/+ Lepr^db^/J male mice (LEPR) received by intraperitoneal of two doses of 150 mg/kg for SJL/J mice and 100 mg/kg for the other strains of ENU (Sigma-Aldrigh, St Louis, MO) a week apart. After 8 weeks of infertility, ENU injected mice (G0) were bred with their isogenic background females to generate G1 offspring. The G1 mice were screened for various phenotypes (abnormal blood count, proteinuria or resistance to rodent malaria infection). Phenodeviant G1 mice were crossed with C3H/He or C57BL/6 mice and progeny tested for their heritability. All mice in this study were housed under controlled temperature (21 °C) with a 12:12 hr light-dark cycle. All procedures were conducted in accordance with the policies of the Macquarie University and conformed to the National Health and Medical Research Council (NHMRC) Australian code of Practice. The work was performed under the agreement Ethics ARA 2012/019 and ARA 2014/55 approved and obtained from the Animal Ethics Committees at the Australian National University and Macquarie University.

### Sequencing

DNA was collected from tails and extracted using a Qiagen DNAse Easy blood and Tissue Kit (Hilden, Germany) according to the manufacturer’s instructions. Ten micrograms of DNA was prepared for paired-end genomic libraries using a paired-end preparation kit from Illumina (San Diego, Ca), following the manufacturer’s instructions. Exome enrichment was performed using an Agilent Sure Select or Nimblegen mouse Exome kit according to the manufacturer’s protocol. Enriched libraries were tested for enrichment using quantitative PCR. The samples were then sequenced on an Illumina Hiseq 2000 platform, which generated paired-end reads of 100 nucleotides. The libraries were multiplexed and barcoded. The exome coverage was 50 × on target.

### Variant calling

Calling mutations from raw sequencing data (fastq) is done by combining the result from four separate mapping and variant calling pipelines (see Additional file 1: Figure S17) to avoid loosing variants due to algorithm bias. All pipelines are executed in the NGSANE [14] framework for reproducible analysis on high performance compute infrastructure. Note, where not stated otherwise, the default settings were used. P1 and P2 uses BWA V0.6.1 [15] and BOWTIE2 [16] V2.1.0 in conjunction with SAMTOOLS [17] V0.1.19 to call variants (mpileup -q1 -D) in each offspring-groups individually after removal of duplicates (rmdup) and subsequent filtering (vcfutils.pl-D1000 -w0 -e0). P3 and P4 also map with BWA but in addition use GATK [18] 2.5 to realign reads (-T RealignerTargetCreator, -T IndelRealigner) and recalibrate the quality score (-T BaseRecalibrator, -T PrintReads) to improve the read location and base-pair call-accuracy. While P3 again uses SAMTOOLS, P4 uses GATK’s UnifiedGenotyper (-stand_call_conf 30.0 -stand_emit_conf 10.0 -dcov 1000) with subsequent hard filtering of variants (-T VariantFiltration, for mutations: – filterExpression “MQ0 ≥ 4 && ((MQ0 / (1.0 * (DP+1))) > 0.1)” and for mutations and indels: –mask [called indels] –clusterWindowSize 10 –filterExpression "QUAL < 30.0 || QD < 5.0 || HRun > 5 || SB > -0.10") to call variants over all mouse genomes in the study simultaneously. In all cases, reads were mapped against Genome Reference Consortium Mouse Build 38 (GRCm38, equivalent to UCSC mm10). The known mouse variants used during realignment, recalibration and GATK variant calling were downloaded from UCSC (dbSNPv128, mm9) and lifted-over to mm10. Only variants in the targeted regions as provided by Agilent (lifted over from mm9) were considered.

### ENU variant filtering

We group the offspring genomes by founder and compare the identified mutations to those from other founder-groups. Only investigating mutations that occur in all offspring from one founder (concordant) and not in those from other founders (exclusive) will robustly separate segregating ENU-triggered mutations from strain-specific variants or sequencing errors. After variants are available from P1-P4 we combined the information from all founder-groups using GATK (-TCombineVariants) and annotated them using GATK (-TVariantAnnotator) with dbSNPv128 and variants from Sanger’s The Mouse Genomes Project (lifted over from mm9). We then retained only complete and exclusive variants that have not been annotated previously (dbSNP, Sanger) using in-house python scripts. These scripts also filtered out variants that were only supported by one method (P1-P4) as well as specific genotypes (homo- or heterozygous mutation) that could be ruled out based on prior genetic information. The resulting high-confidence variants were then functionally annotated using ANNOVAR [19] (Ensembl, MGI, dbSNP137, phastConsElements60way) and variants overlapping olfactory genes were removed.

### Open chromatin information

DNase-Seq library was downloaded from GEO (GSE53076, bed-files) for Pre-Sertoli cells from the testes of E15.5 mouse embryos (Sox9 Heterozygous male embryos conceived from transgenic homozygous Sox9-ECFP C57BL/6J male mice mated to female CD-1 (Charles River) females) as well as heart, brain, liver, kidney and fibroblasts. Chip-Seq data for testes, heart, liver, kidney for histone marks H3K27ac, H3K27me1, H3K4me1 was downloaded from Encode Experiment Matrix (broadpeak). The areas of active regions are generated by concatenating the genomic regions from H3K4me1 and H3K27ac and removing the areas containing Chip-Seq peaks from H3K27me1 using BEDTOOLS [20].

## Additional files

Additional file 1
**Main supplemental file.** Additional figures and tables. (PDF 1720 kb)

Additional file 2
**Genes potentially not covered by germ line ENU approaches.** MGI names and location of genes overlapping H3K27me3 histone marks, which are considered closed genomic areas and hence potentially not targeted by ENU mutations that need to be passed on through the germ line. (TXT 379 kb)

## Abbreviations

DNase-Seq: DNase hypersensitivity sequencing data
ENU: N-ethyl-N-nitrosourea
LEPR: BKS.Cg-Dock7m +/+ Leprdb/J male mice
SNP: single nucleotide polymorphisms
ti: transition
tv: transversion
UTR: untranslated region
