# Supplementary material for: Genome-wide analysis of chemically induced mutations in mouse in phenotype-driven screens
Source: BMC Genomics. 2015 Oct 26;16:866. doi: 10.1186/s12864-015-2073-4 (PMC4623266; doi:10.1186/s12864-015-2073-4)
Supplement: Additional file 1 — Main supplemental file. Additional figures and tables. (PDF 1720 kb) [file 12864_2015_2073_MOESM1_ESM.pdf]

Mapping and ENU mutation statistics

Extended mapping statistics in Table 1, number of ENU mutations per founder in Table 2. Visualisation of ENU mutation and read coverage per founder, background, provider and capture technology in Figure 1 and Figure 2 respectively. Figure 3 shows the mean genomic coverage per founder, background, provider and capture technology. Figure 4 and Figure 5 correlates the number of mutation and general genomic coverage against the mean coverage at the ENU sites.

Table 1 Mapping statistics

Bwa Statistics	Total reads	Duplicates (%)	Mapped (%)	Paired (%)	Mean coverage	% bases covered >25
min	13216116	1391178 (2.8)	13090628 (86.9)	12920714 (81.1)	13.6	18.5
max	173767104	31303294 (39.4)	165721692 (99.1)	157090136 (97.8)	174.3	95.2
mean	74755975	14663961 (20.4)	70783797 (94.8)	67350409 (90.4)	53.3	75.0
ste	2531660	729818 (1.0)	2378946 (0.2)	2224692 (0.3)	20.5	11.0
Bowtie2 Statistics	Total reads	Duplicates (%)	Mapped (%)	Paired (%)	Mean coverage	% bases covered >25
min	13216116	1372143 (3.0)	13151902 (92.1)	12738554 (86.7)	13.3	18.3
max	173767104	32739770 (39.5)	169023408 (99.5)	161866664 (97.6)	181.3	95.2
mean	74755975	14639249 (20.4)	73136560 (97.9)	69670164 (93.3)	56.2	77.4
ste	2531660	731334 (1.0)	2459273 (0.2)	2346902 (0.2)	21.6	10.5

Table 2 Mapping statistics

Background	Number of Founder	Mean ENU mutations
BL6	9	11.0625
(C3H/HexSJL)F1	4	8.000
LEPR	2	17.500
SJL	40	28.175

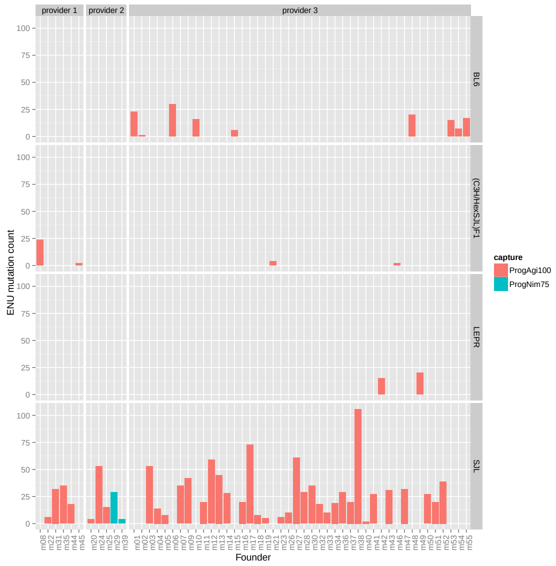

Figure 1 ENU count per background, provider and capture technology.

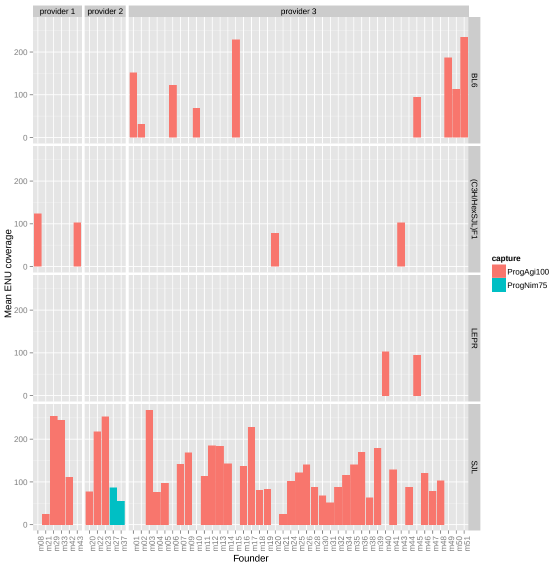

Figure 2 Mean coverage at ENU sites per background, provider and capture technology.

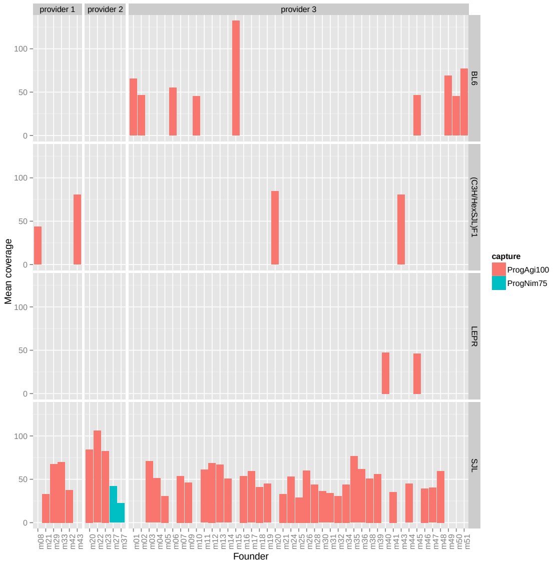

Figure 3 Mean genomic coverage per background, provider and capture technology.

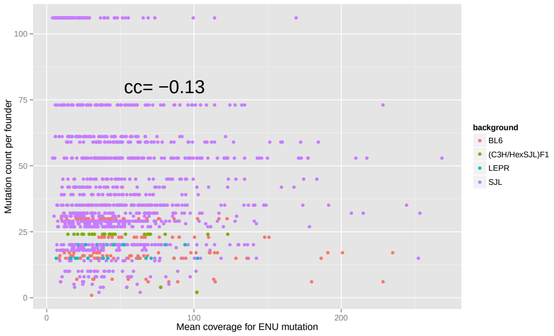

Figure 4 Number of ENU mutations vs mean coverage.

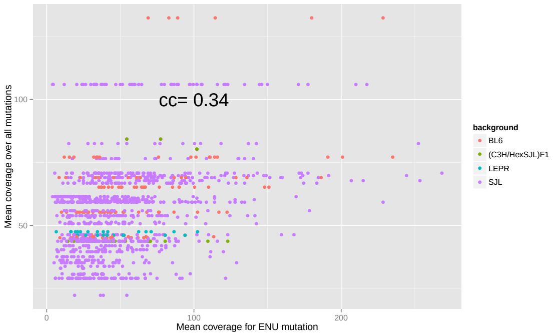

Figure 5 Mean coverage of ENU mutation vs mean coverage over all mutations

Substitution frequency

We count the different substitutions for the G1 as well as mutagenetix and phenomics datasets.

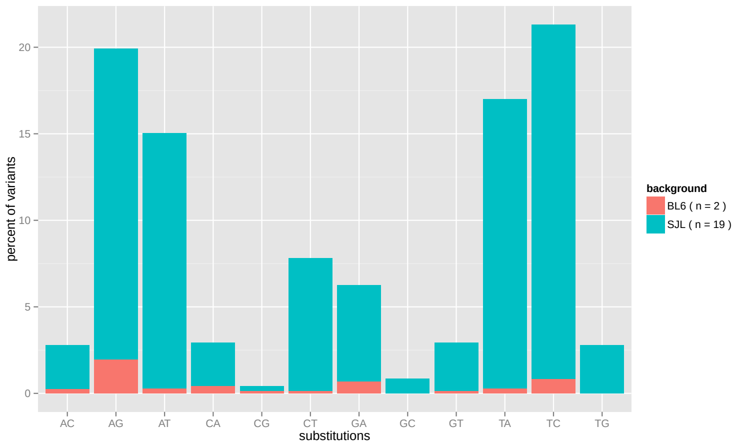

Figure 6 Substitution frequency for G1 only.

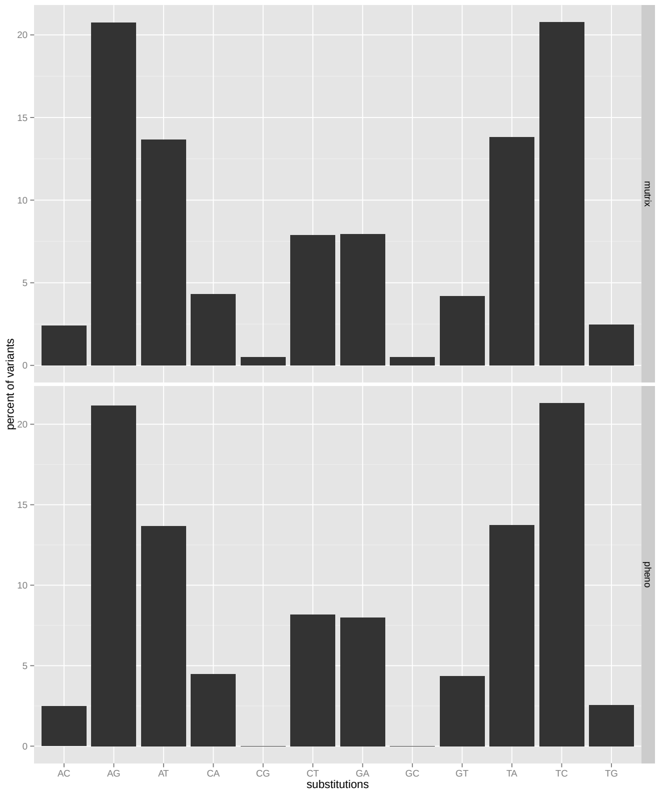

Figure 7 Substitution frequency for mutagenetix and phenomics.

ENU motifs

We called strain specific variants using BOWTIE2 on sequence data from unmutated mice with SJL and (C3H/HexSJL)F1 background. We then modify the reference genome, GRCm38, with strain specific variants using an in-house script and extracted the sequence of up to 10bp flanking the ENU mutations of SJL or (C3H/HexSJL)F1 founders. We visualise the resulting data using WebLogo [1].

Figures

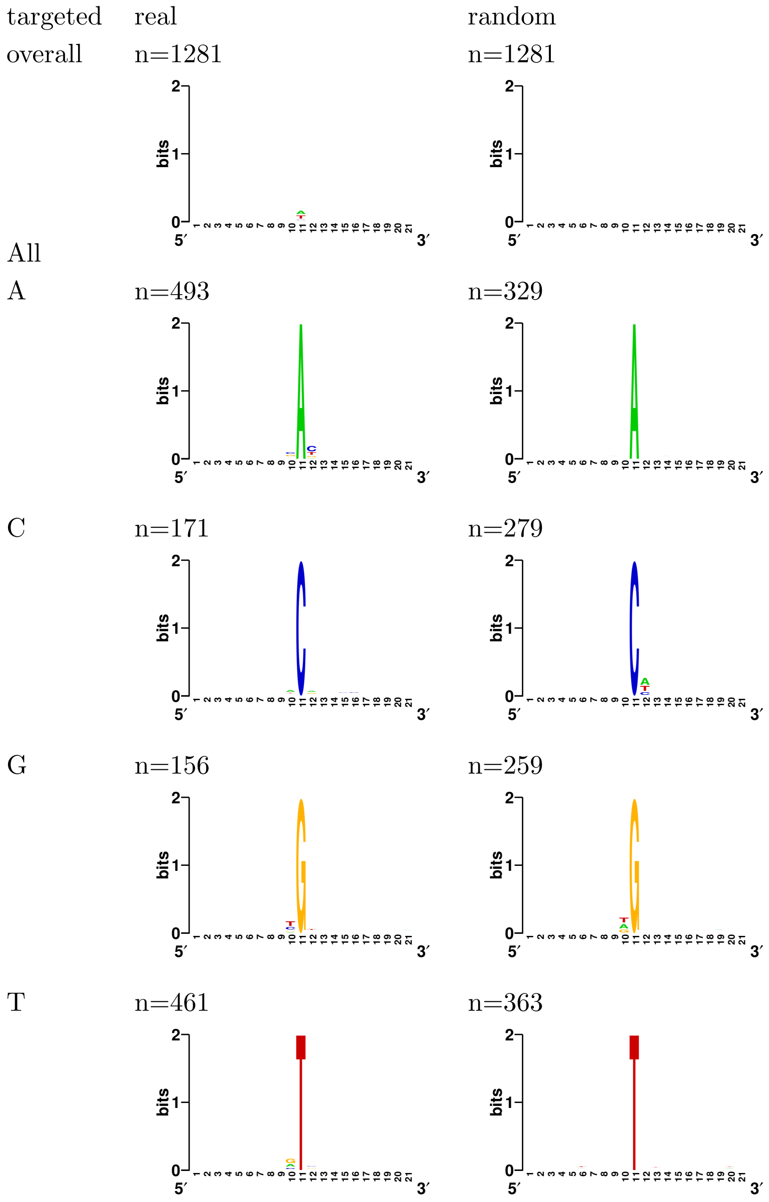

Figure 8 **Motif for All.**

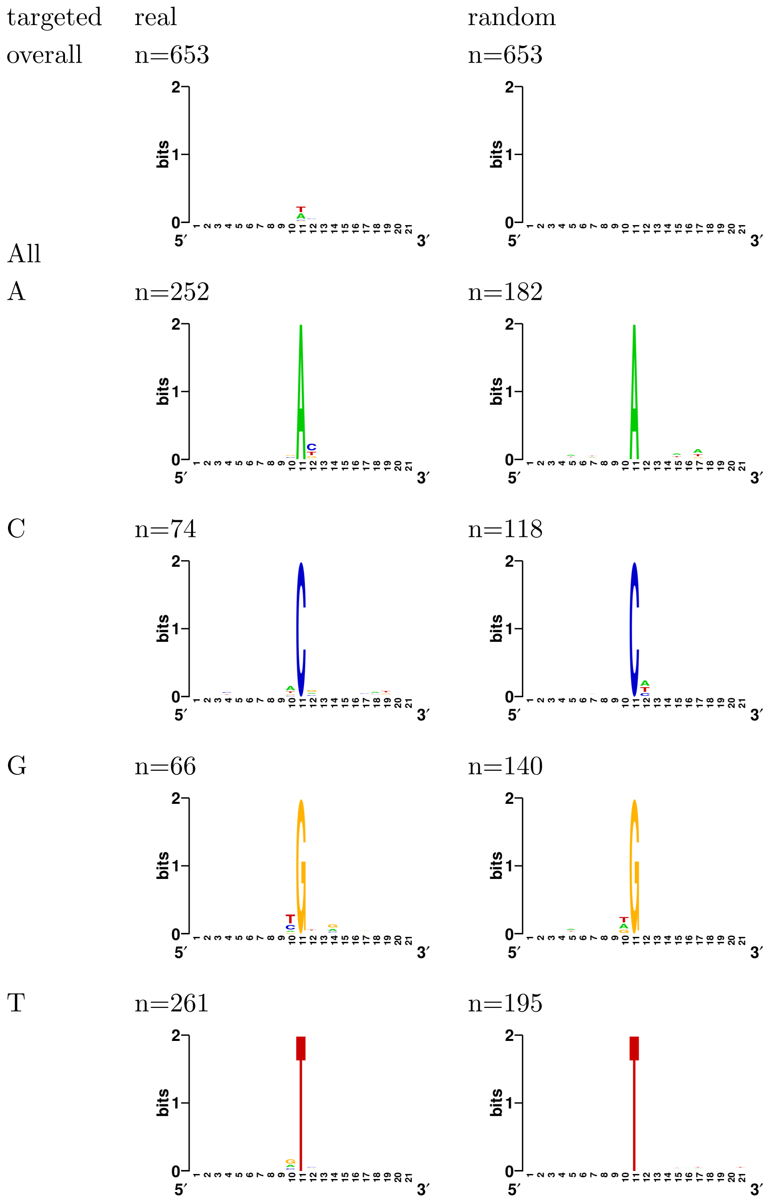

Figure 9 Motif for G1.

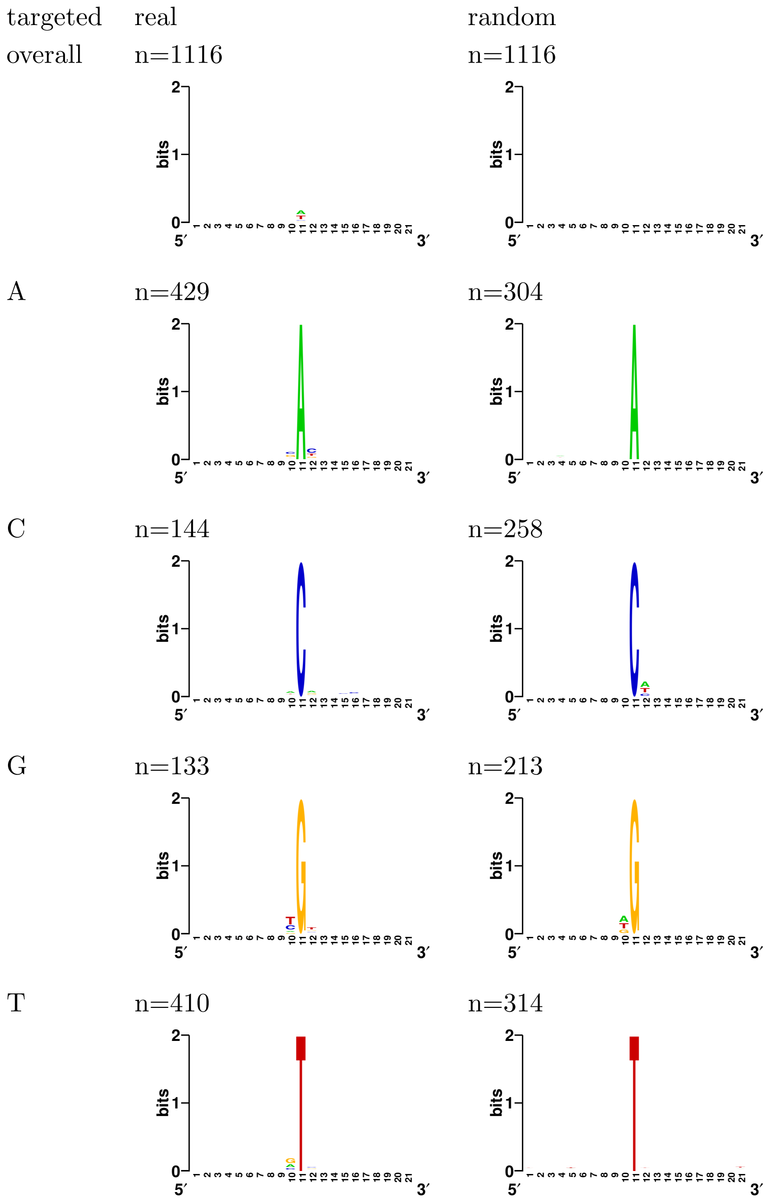

Figure 10 Motif for SJL.

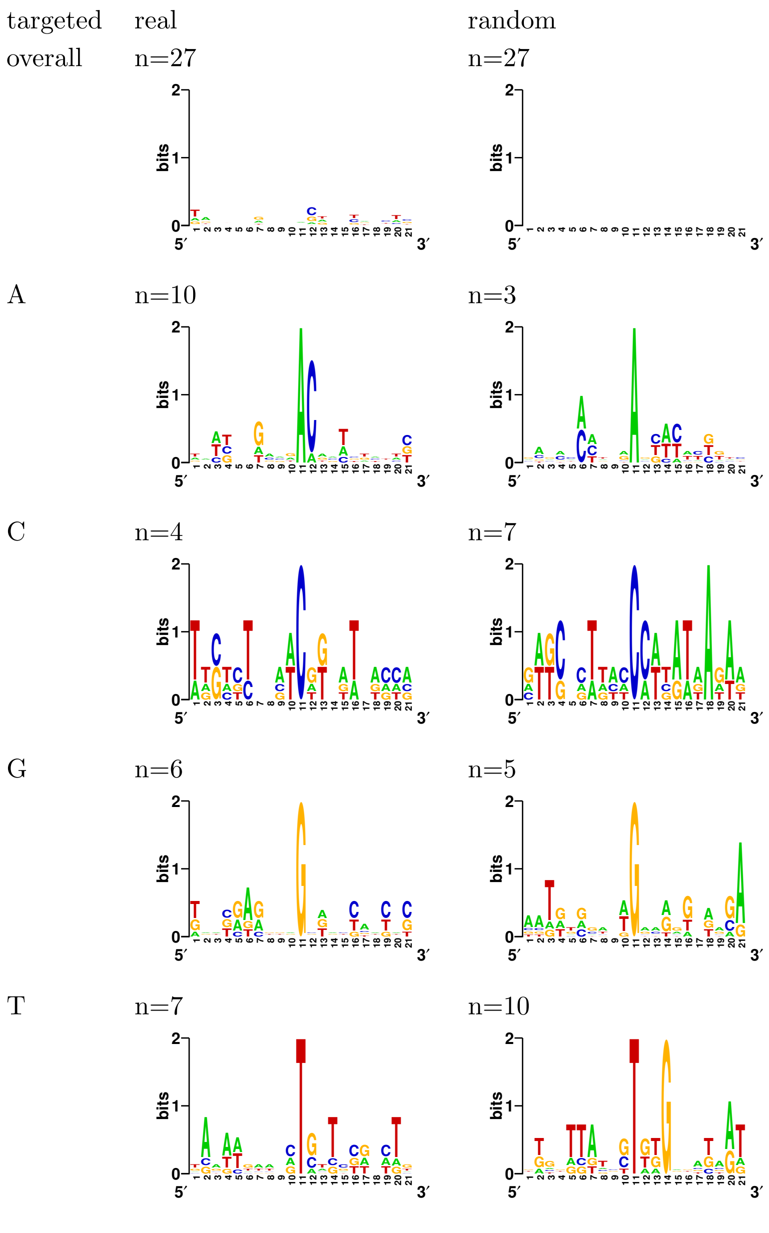

Figure 11 Motif for (C3H/HexSJL)F1.

Distribution in genome

Figure 12 shows the location of ENU mutation in the G1 generation colour coded by founder. Figure 13 and Figure 14 show the location of all ENU mutations reported in the phenomics and mutagenetix dataset, respectively. Figure 15 shows the ENU mutation location per founder. Figure 16 shows a histogram of targeted genes for the real ENU mutation as well as randomly selected locations in the genome or exome (1000 times).

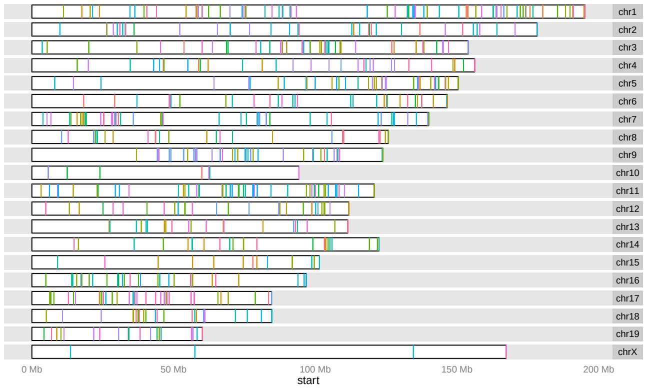

Figure 12 Distribution in genome G1.

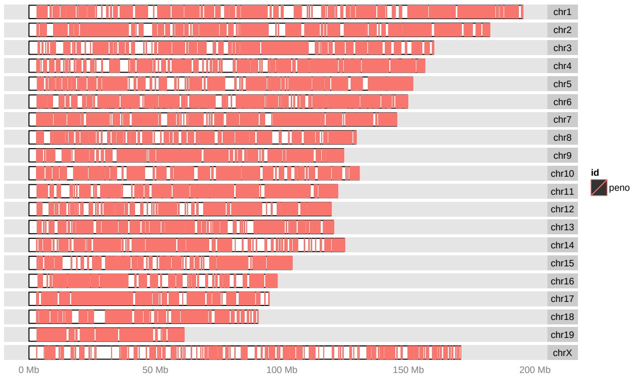

Figure 13 Distribution in genome phenomics.

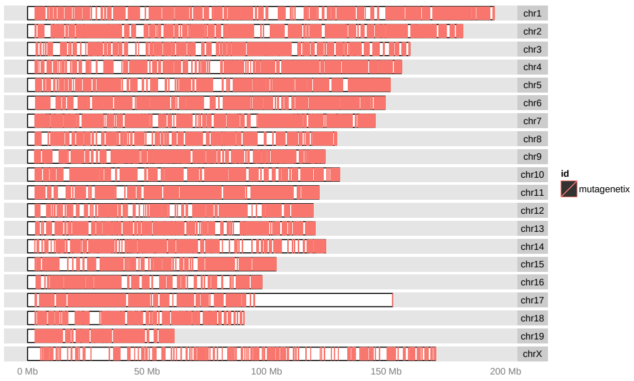

Figure 14 Distribution in genome mutagenetix.

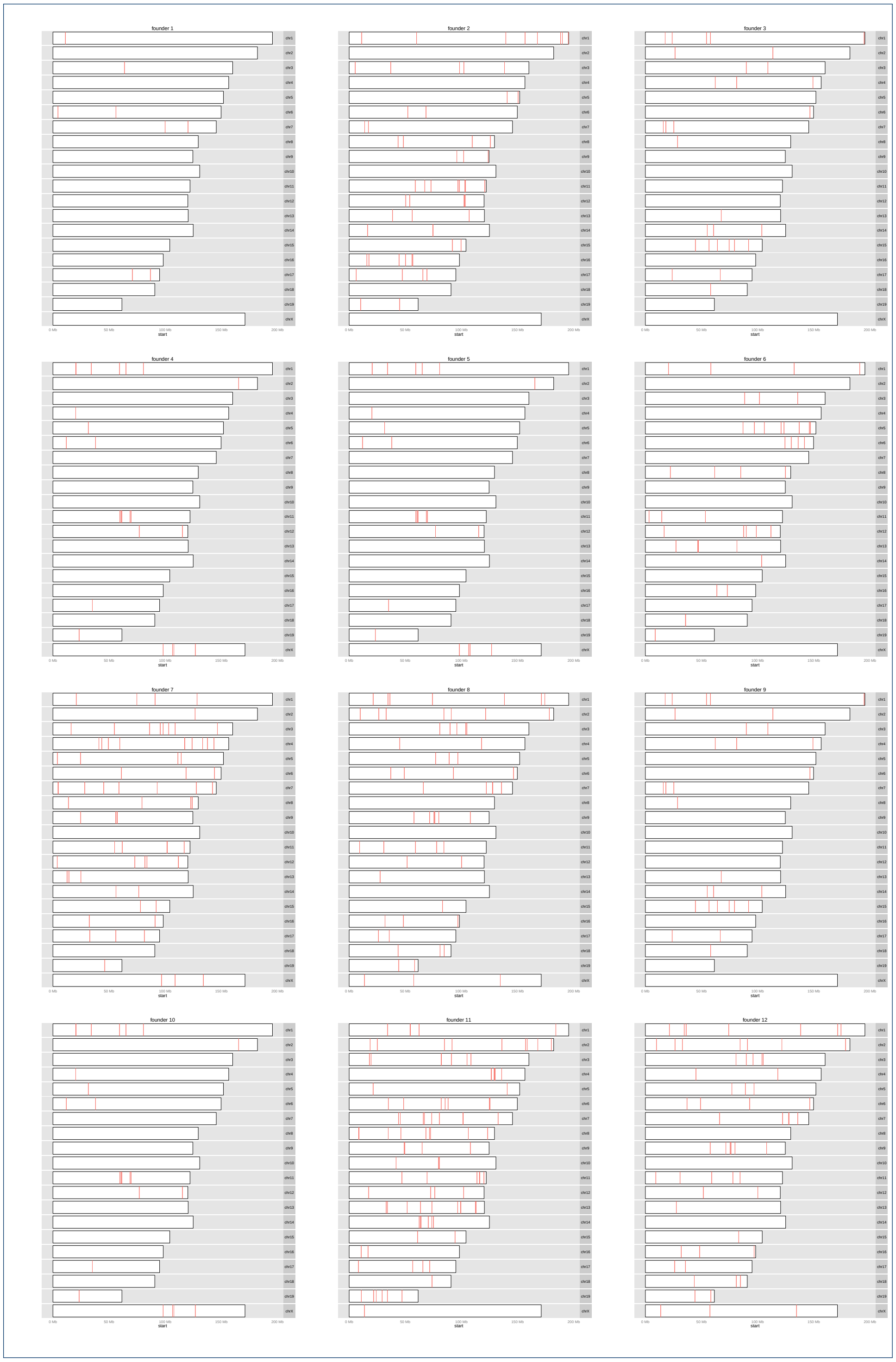
founder 1
founder 2
founder 3
founder 4
founder 5
founder 6
founder 7
founder 8
founder 9
founder 10
founder 11
founder 12
chr1
chr2
chr3
chr4
chr5
chr6
chr7
chr8
chr9
chr10
chr11
chr12
chr13
chr14
chr15
chr16
chr17
chr18
chr19
chrX
0 Mb
50 Mb
100 Mb
150 Mb
200 Mb
start

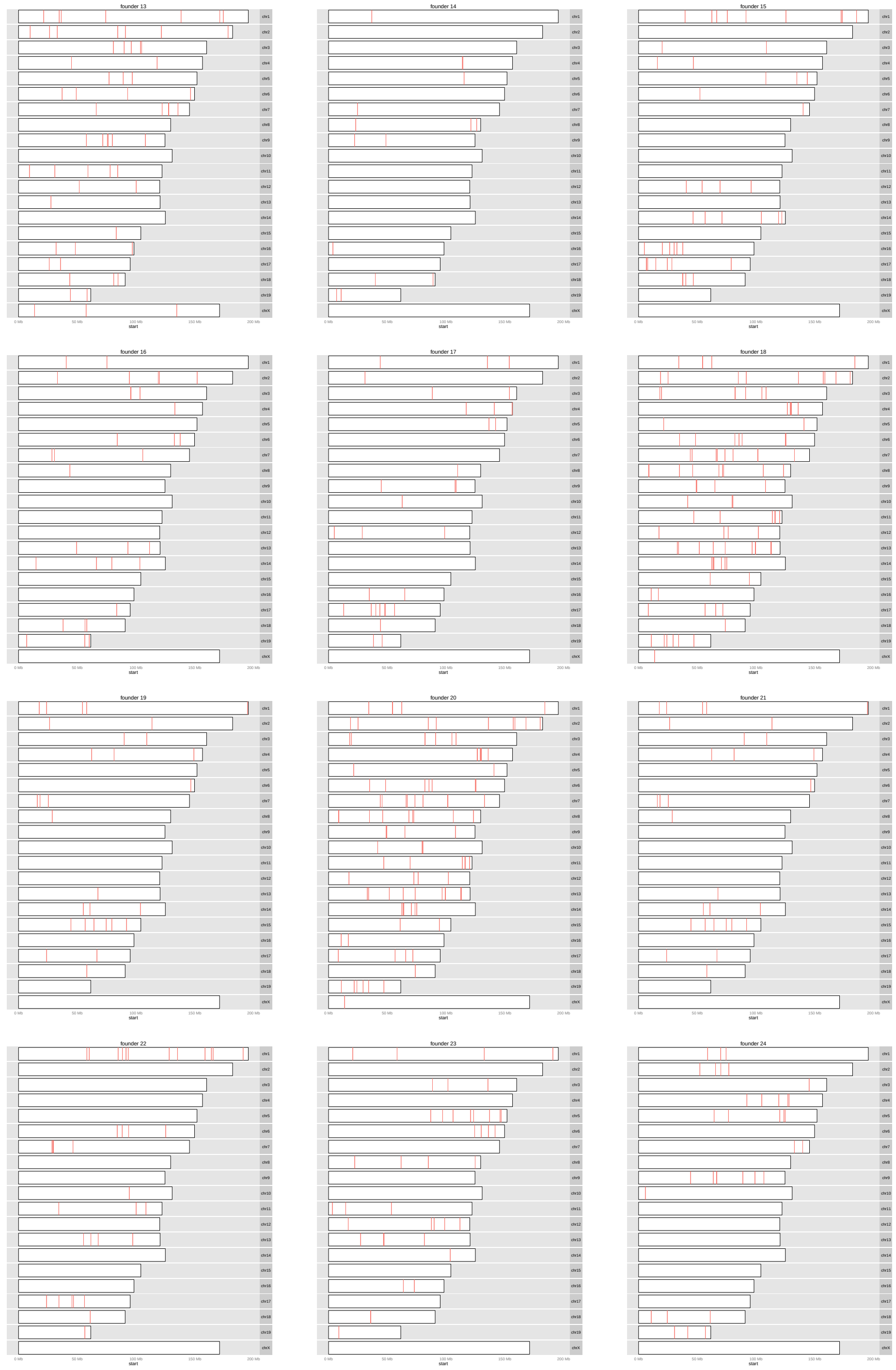
founder 13
founder 14
founder 15
founder 16
founder 17
founder 18
founder 19
founder 20
founder 21
founder 22
founder 23
founder 24
start

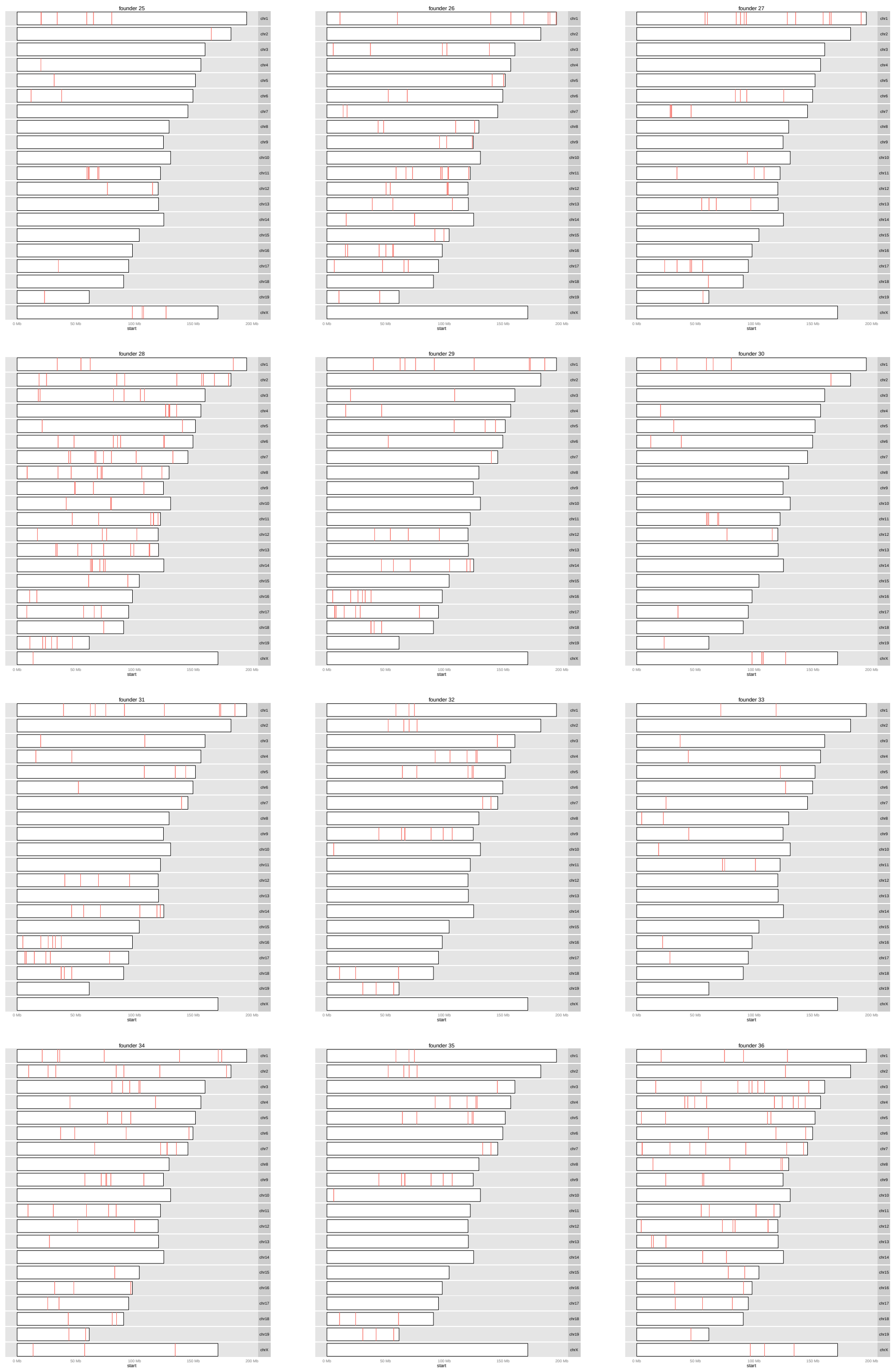
founder 25
founder 26
founder 27
founder 28
founder 29
founder 30
founder 31
founder 32
founder 33
founder 34
founder 35
founder 36
chr1
chr2
chr3
chr4
chr5
chr6
chr7
chr8
chr9
chr10
chr11
chr12
chr13
chr14
chr15
chr16
chr17
chr18
chr19
chrX
0 Mb
50 Mb
100 Mb
150 Mb
200 Mb
start

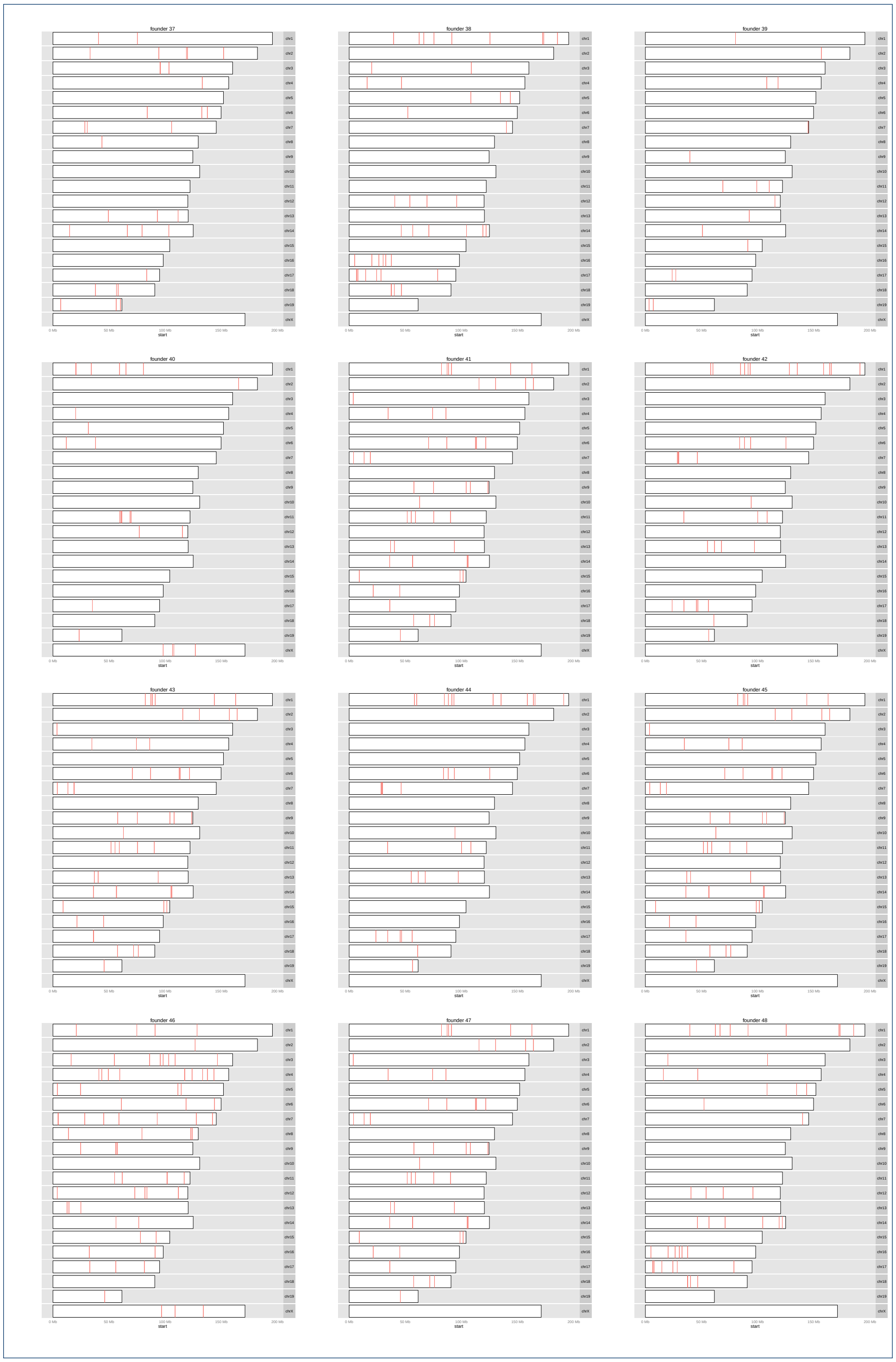
founder 37
founder 38
founder 39
founder 40
founder 41
founder 42
founder 43
founder 44
founder 45
founder 46
founder 47
founder 48
start

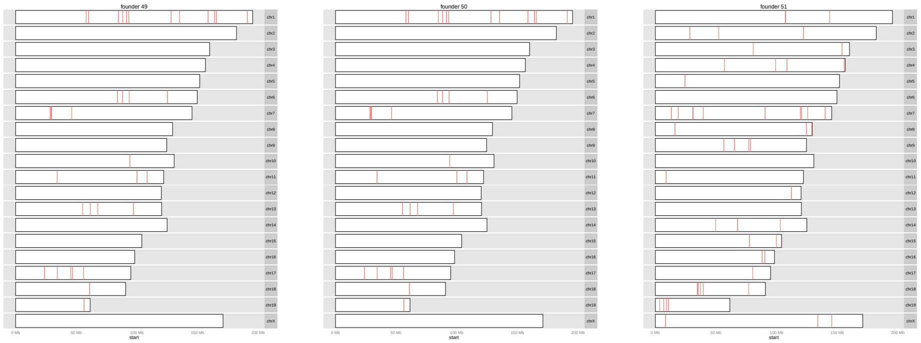

Figure 15 Distribution in genome mutagenetix.

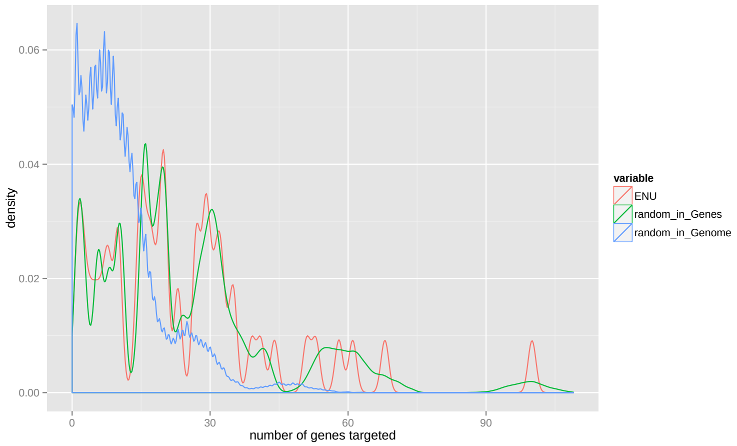

Figure 16 Distribution of targeted genes

Method

Figure 17 is a visual representation of the pipeline and Table 3 shows the number of genes and their properties that are reported to contain an ENU mutation.

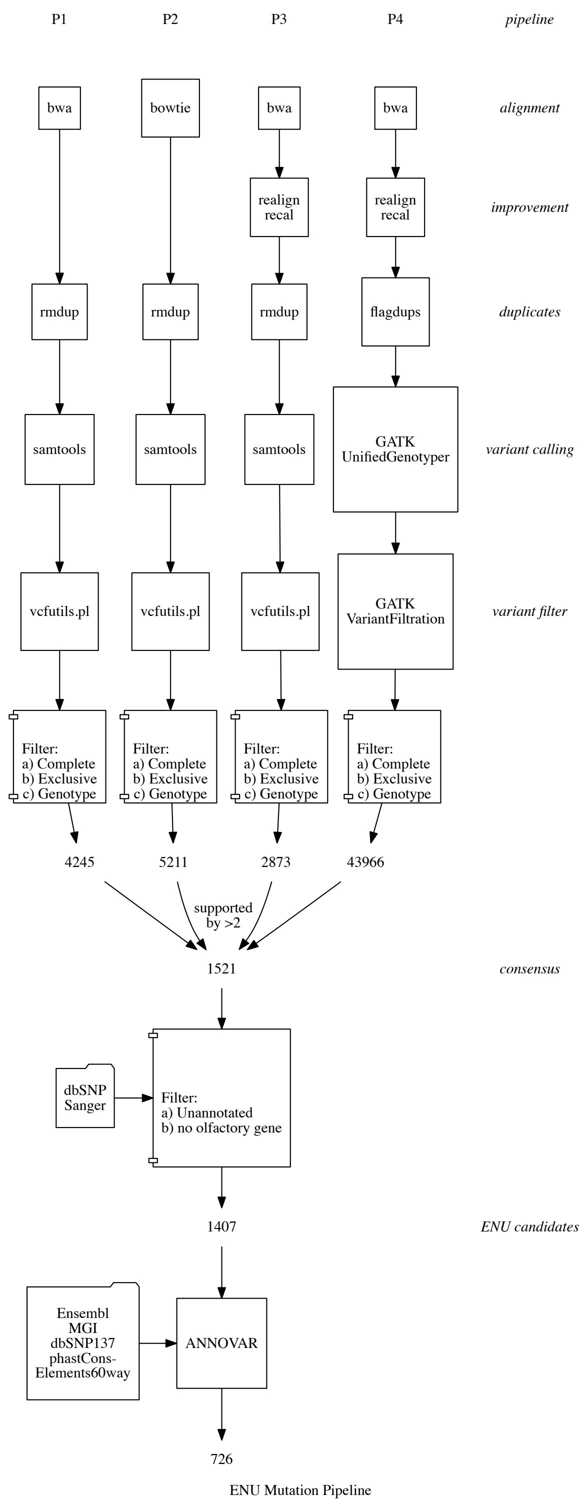

Figure 17 Pipeline shows the pipeline components

Table 3 **Mean of GC and gene length** Note, not all identified variants are located in annotated genes

	number of genes	CG	gene length
mouse genome	43629	46.1602	26550.38
ENU all	1186	45.6114	88498.69
ENU G1	613	45.1885	94356.03
mutagenetix	19491	46.1220	49908.29
phenomics	20496	46.1544	48533.53

Author details

References

1. Crooks, G.E., Hon, G., Chandonia, J.-M., Brenner, S.E.: Weblogo: a sequence logo generator. Genome Res **14**(6), 1188–1190 (2004)
